# Supplementary material for: Identification of Freezing-Responsive microRNAs and Their Targets in Chinese Jujube by Small RNA and Degradome Sequencing
Source: Int J Mol Sci. 2026 Sep 4;27(17):7912. doi: 10.3390/ijms27177912 (PMC13566890; doi:10.3390/ijms27177912)
Supplement: Supplementary file 1 [file ijms-27-07912-s001.zip › Supplementary Material S2/novel_miR_137.pdf]

Provisional ID	:	novel_miR_137
Score total	:	0.5
Score for star read(s)	:	-1.3
Score for read counts	:	0
Score for mfe	:	0.2
Score for randfold	:	1.6
Score for cons. seed	:	
Total read count	:	56
Mature read count	:	56
Loop read count	:	0
Star read count	:	0

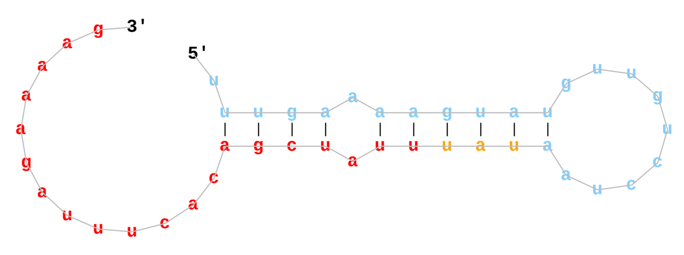

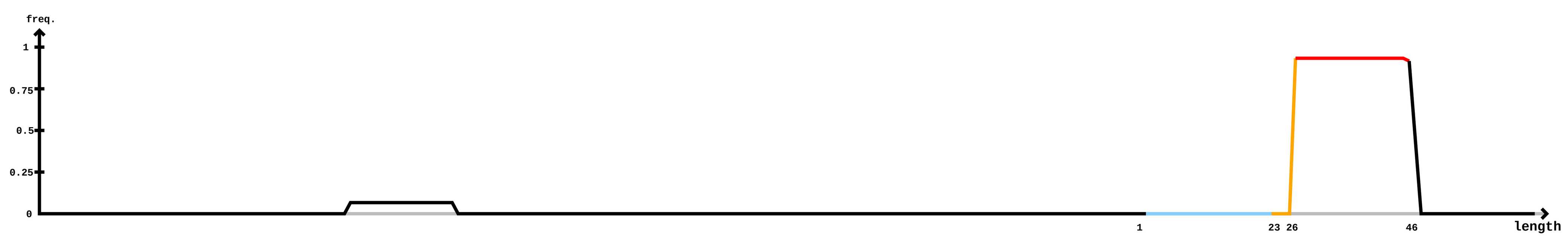

Star Mature

```
5'- guuauaaaaauauaaguacgaggauauccggcuguaauaaaauaaaaccucaagagugugcaguguaauauacccuaaaaaaaaguaauagacgaugcuuuuucgaaaaagcgucgcccuuuuuaccacauuuagugacccuuauucagaaaagcgucaccuauucuaacauuuagcgaugcuuuugaaaaguauguuguccuaauauuuaucgacacuuuagaaaagcaucgcuuauuuuuaguaucuag -3'   exp
    ..............((((..((((((...((((((.....................))))))...)))).)).........((.(((((..((((((((((.(((.(((.(((((................))))).))).))).))))))))))..)))))))......((((((((((((..(((((..((((.............)))))))))..))))))))))))......))))....
```

read	reads	mm	sample
caagagugugcagugAaa	2	1	JCK-2
caagagugugcagugAaa	2	1	JCK-1
uuaucgacacuuuagaaaU	1	1	LT-3
uuaucgacacuuuagaaaag	27	0	LCK-2
uuaucgCcacuuuagaaaag	1	1	LCK-2
uuaucgacacuuuNgaaaag	1	1	LCK-1
uuaucgacacuuuagaaaag	26	0	LCK-1
